# Pennsylvania State Dental Society

**Published:** 1889-12

**Authors:** 


### Reports of Society Meetings.

### PENNSYLVANIA STATE DENTAL SOCIETY.—(*Concluded.*)

*Wednesday, July 31.—Afternoon Session.*

### DISCUSSION ON DR. ROBERTS'S PAPER.

*Dr. Beck.*—I would like to ask the essayist regarding the resistance machine, White's manufacture, whether it is necessary to use three lamps of sixteen candle-power to bring down the power to sufficient strength to run the mallet?

*Dr. Roberts.*—One lamp of sixteen candle-power takes about seven-tenths of one ampère. Three lamps would represent about two ampères of current. The current must be reduced that much; otherwise it will burn the mallet out. Then we have also to reduce the voltage of the current, which is done by shunting part of the current through the mallet, the resistance of the mallet being a great deal more than the rest of the circuit for which it acts as a shunt. They use a very small part of the current that goes through. I think a better arrangement could be made.

*Dr. Beck.*—Dr. Starr has demonstrated it to me, and it occurred to my mind that in using three lamps of sixteen candle-power it used a vast amount of power to reduce the amount down to drive the mallet. That power on the mallet would burn the mallet out, and I asked him the same question,—Whether two lamps of the same capacity would not give the same results?

*Dr. Roberts.*—He uses the lamp because it is the cheapest kind of resistance. He claims to use the heat that would be wasted in any other way, which I think is good. Another way of using the mallet on the incandescent current, I think, would be serviceable and a better arrangement. It is to make a motor, winding it with two independent coils on the armature, one with a few turns of wire and the other with many. The motor can be made so that it will require less than one ampère, or the current that goes through one lamp, to run it. The armature of the motor would have a commutator at both ends, one for each of its independent coils. The mallet would be connected to the commutator attached to the coil having a few turns of wire, and the motor placed in the main circuit. When the motor is run, there would be generated a current in the coils connected with the mallet, the potential of which would be in the same proportion to the main circuit as the number of turns of wire in the mallet coils are to the number of turns in the motor coils. If there were one hundred turns in the motor, and ten turns in the mallet coils, there would be only one-tenth the pressure through the mallet that there would be in the main circuit, and in that way you can get the potential down to almost any amount. The same motor could also be used to drive the engine, and when you were not using the mallet the brushes would simply be raised so as not to touch the commutator.

*Dr. Beck.*—I went to our electrical company, who runs the two kinds of power, arc and incandescent, and I saw them putting in dynamos for various kinds of power, and it occurred to me that they might make a resistance box so as to run the mallet by taking the power from incandescent wires. They said they could not form it in that way,—that they would be afraid to introduce it to drive so delicate an instrument as the mallet. All of you who run an electric mallet, and who use a Bunsen battery, know what a nuisance it is to charge the battery. I use four cells, and sometimes a fifth, but I have to alternate these cells. Mostly, if I have a large amount of heavy work, I have to renew two cells alternately during the week. If we can get power from the incandescent lamp which will do away with all this changing of cells, it will be an important advance.

*Dr. Faught.*—I am glad Dr. Beck spoke of the battery, also the mention he makes of the hot-air apparatus. It is a very good one. To those who are compelled to use a battery I would say that from personal experience there is no better battery than the Partz. It is a sulpho-chromate battery. With it I run my mallet; also my lamp for heating purposes, and the very best average is only once in three months does it require cleaning, when you have only to pour out the fluid, wash it out with water, and fill it up again. There are no dirty acids or salts deposited anywhere about the parts. It is easily filled, and acts immediately. The fluid is made from a patent salt. I can do all the work I want to, and in about three months, if the battery gets weak, I add about a spoonful more salt, and it will be sufficient for several weeks more.

While on the subject and uses of electricity, I want to mention a little instrument of my own invention that will shortly be put on the market. It is to be used for filling teeth with gutta-percha. It consists of a handle, to which points adapted to filling any cavity can be attached. These points can be heated to any desired temperature by means of electricity passed through the handle to a resistance material situated at the base of the point.

*Dr. Beck.*—Could not an instrument of this kind be made for annealing gold, instead of the use of the lamp?

*Dr. Roberts.*—It would depend upon the weight of the gold how much current would be required. That would be regulated by a resistance coil. The gold would simply be placed on the conductors to heat the full length, or mica or some such substance could be heated, by a current passing on one side of it, to any degree, and the gold laid upon the other side to be annealed. Then it would make no difference how heavy the gold was.

*Dr. Magill.*—There was another question: What have you to say in regard to the danger?

*Dr. Roberts.*—I think the danger is more imaginary than real. The day before coming here I placed my hand on two live wires. You cannot ground the current of the incandescent wire, because there is not enough voltage to overcome the resistance. The arc-light wire would be very dangerous. The danger is through the ground, and you cannot take hold of an arc-light wire. With the incandescent light you can burn yourself to a blister, and that is all you can do without a special effort, and the patient is not going to sit quiet long enough to be burned.

*Dr. Magill.*—When both instruments are in use in the same vicinity, can there be a possibility of transfer or interchange of arc and incandescent current?

*Dr. Roberts.*—It is so arranged that only a given amount of current can pass through. A wire of this kind will seldom carry over two ampères of current. It will burn or melt, and break the circuit.

*Dr. Green.*—Do I understand that, in taking four cells, the power of the battery is represented by the two weak, or the two strong cells?

*Dr. Roberts.*—There would be a balance or average between the stronger and weaker cells. Where the cells are arranged in series, the carbon in one to the zinc in the next, you get the greatest pressure. If you connect all your carbons together you have simply one large cell. If the resistance of the mallet is such that enough current will run through to run the mallet, that would be the most economical arrangement you could get; but the mallet is not wound that way, for the resistance there is so great that there must be more than two volts to get it to run well.

*Dr. Green.*—I think we ought to be very grateful to Dr. Roberts for giving us such a distinct definition of the various terms used at the present day. We may take this entire assembly, and not more than a few of us can define the terms. The manner in which he has brought the subject before us, I think, is deserving of a great deal of attention. I now feel that light has been thrown upon much that has been dark to me in the proper understanding of the terms used by electricians. Now that electricity is to be used for executions, and is being introduced in various places as a means of power, it is very important to look the matter squarely in the face.

He tells us that out of all the coal we burn in an engine there is not more than ten or twenty per cent. converted into energy. Think of the amount of coal we are throwing away,—getting only about six per cent. of service out of the entire body. It is natural for men to look towards some other means for getting power, and the doctor has very nicely presented the subject as to the advantages of the electrical power over some others now in use.

*Dr. Roberts.*—Regarding executions, I do not believe that criminals will ever be executed by electricity. It can be, but will not be. There is too much opposition to it; and another thing, those men who manufacture generators, or machines to give the incandescent current without using transformers, say, "Take my dynamo and execute with it if you can." On the other hand, the arc men are sending men on there to add their testimony that it is not safe without ascertaining what the resistance is. It is possible to make such connections with street wires—the incandescent—that you can only get in your office a certain number of volts. They will not do it, because it is out of their line. If we have a wire split and brought together again at the other end, either wire could be called a shunt to the other,—it divides and comes together again. Any current that leaves the dynamo must come back to it in some way. The S. S. White Company claim to reduce the potential by shunting most of the current around the mallet. I should suppose that the resistance of the mallet and the shunt are in the same proportion to each other as the voltage they want through the mallet is to the voltage of the main circuit.

*Dr. Beck.*—I do not know whether there is any difference in the force, but I want to say this regarding this point. We have two electrical companies in our place,—one incandescent, one arc. Then we have a street railway which runs an electric car. By some means a locomotive passing along broke the overhead wire over the car which makes the connection, and before it could be taken up a horse came along, and as soon as he put his foot on the wire he dropped dead. Another peculiar occurrence was that in a heavy thunder-storm a dynamo ceased to work, and upon examination they found a bolt had struck a wire somewhere. The dynamo was taken apart to ascertain the cause of its stopping. I understand the dynamo afterwards had to be recharged.

*Dr. Roberts.*—The wire will only carry a definite amount of current. The bolt of lightning which struck the wires sent the current through the dynamo in amount greater than the dynamo would carry. It burnt out the insulation.

*Dr. Beck.*—In ascending high hills there is no difference in the momentum of the cars. Conductors tell me that when these cars are heavily loaded they find a slight difference in the velocity of the car, but the car goes up the hill the same as on a level.

*Dr. Faught.*—While the subject of the car is under discussion, I think if you draw your circuit from the same wire that manages these cars there will be no satisfaction to a dentist, for it will depend upon where the cars are for your light. Then the power will not be regular, and it will also depend on the number of cars on the circuit.

*Dr. Roberts.*—The current they use for running electric cars runs away up from two hundred and twenty to five hundred volts. I think it is possible to get a regulation for the motor to run the engine in a manner so that when you are using a disk and want slow speed, you can get slow speed with the power. With the arrangements they generally use now, when you get slow speed there is no power. It is theoretically possible to make a motor so that when you are running it slowly you will get more power than when running it faster. Motors are not made in that way, because there is no demand for them, but if I can I want to perfect it.

*Dr. Klump.*—Is the difference in capacity due to the difference in resistance or the winding of the wire?

*Dr. Roberts.*—The difference in capacity is due to the resistance, the winding, and the counter-electromotive force generated by the motor.

Subject passed.

